# Just culture and its influence on nurse interns’ willingness to report near-miss events: a cross-sectional study in five Egyptian university hospitals

**DOI:** 10.1186/s12912-025-03979-w

**Published:** 2025-11-13

**Authors:** Mustafa Mohamed Dwidar, Samah Faisal Fakhry, Laila Ahmed Abdel-Hamed

**Affiliations:** Faculty of Nursing, Ain Shams University, Cairo, Egypt

**Keywords:** Just culture, Near-miss reporting, Nurse interns, Patient safety, Organizational culture

## Abstract

**Background:**

Near-miss incidents represent critical learning opportunities in healthcare, yet they are frequently underreported due to fear of blame and lack of organizational support. A Just Culture framework promotes fairness, trust, and learning from errors, which may encourage voluntary reporting, especially among nurse interns in high-stakes clinical environments.

**Aim:**

To assess the perception of Just Culture among nurse interns and examine its influence on their willingness to report near-miss events in governmental university hospitals in Egypt.

**Methods:**

A cross-sectional analytical study was conducted among 163 nurse interns (sampling frame *N* = 264) using systematic random sampling across five university hospitals in Cairo, Egypt. Data were collected between March and April 2025 using a self-administered questionnaire comprising the Just Culture Assessment Tool (JCAT) and a researcher-developed Willingness to Report Near-Miss Scale. Data analysis included descriptive statistics, correlation analysis, and multiple linear regression.

**Results:**

The mean Just Culture score was 3.6 (SD = 0.5), while the mean willingness to report near-miss events was 3.6 (SD = 0.5). Significant positive correlations were observed between Just Culture dimensions (trust, feedback, openness, balance, and continuous improvement) and willingness subdomains (awareness, skills, attitude, behavior) (*p* < 0.001). Regression analysis indicated that Just Culture score (B = 0.70, β = 0.67, *p* < 0.001) was the strongest predictor of willingness to report, explaining 55% of the variance.

**Conclusion:**

A supportive Just Culture significantly enhances nurse interns’ willingness to report near-miss events. Strengthening institutional safety culture through leadership, training, and non-punitive policies is essential to fostering early-career nurses’ engagement in safety reporting.

**Supplementary Information:**

The online version contains supplementary material available at 10.1186/s12912-025-03979-w.

## Introduction

Ensuring patient safety remains a central goal in healthcare delivery worldwide. Despite technological advancements, evidence-based protocols, and quality control measures, the healthcare system continues to be vulnerable to adverse events, many of which are preventable [1]. Central to addressing this challenge is the cultivation of a culture of safety that not only emphasizes procedural accuracy but also fosters an environment where health professionals feel empowered to report errors or near misses without fear of retribution [2]. One foundational concept in the evolution of such a culture is the principle of a Just Culture—a balanced approach that distinguishes between human error, risky behavior, and willful negligence, ensuring that accountability is maintained without resorting to blame-based practices [3, 4].

The concept of Just Culture has gained international attention as an essential strategy for improving organizational learning and mitigating preventable harm. It recognizes that while individuals must be accountable for their actions, most errors result from system failures rather than personal negligence. Thus, a Just Culture promotes open communication, encourages incident reporting, and focuses on learning and system improvement rather than punishment [5]. The adoption of Just Culture principles in healthcare settings facilitates the reporting of adverse events and near misses, enabling institutions to develop preventive strategies, reinforce ethical accountability, and improve patient outcomes [6]. Importantly, Just Culture aligns with the core values of nursing, including integrity, transparency, and a commitment to nonmaleficence [7].

One foundational framework for understanding error and reporting in healthcare is Reason’s Swiss Cheese Model, which conceptualizes adverse events as the alignment of multiple system-level failures with individual lapses, emphasizing that most errors reflect organizational vulnerabilities rather than individual negligence [8]. Building on this systems view, Dekker’s Just Culture model stresses the need for balanced accountability, distinguishing between human error, at-risk behavior, and reckless conduct while fostering a fair environment that encourages reporting and organizational learning [9]. Similarly, the AHRQ Hospital Survey on Patient Safety Culture (HSOPS) provides a structured approach to measuring institutional climate, including trust, openness, and feedback, which are essential precursors for non-punitive reporting [10]. To further explain reporting behaviors, the Theory of Planned Behavior (Ajzen, 1991) offers a useful psychological lens: attitudes toward reporting, subjective norms within the organization, and perceived behavioral control collectively shape the intention to report, which in turn predicts actual reporting practices [11]. Together, these frameworks underscore the importance of fostering a supportive Just Culture to translate nurse interns’ awareness of safety into consistent near-miss reporting.

Near-miss incidents—defined as unplanned events that could have led to patient harm but did not, either by chance or timely intervention—are particularly valuable learning opportunities in clinical environments [12]. These incidents offer insights into latent system vulnerabilities and human factors that, if unaddressed, could result in future adverse events. Despite their importance, near misses are grossly underreported in healthcare institutions, largely due to fear of blame, lack of feedback, and uncertainty about the reporting process [13]. Research indicates that near-miss reporting rates are substantially lower than those for actual adverse events, highlighting a critical gap in safety surveillance [14, 15].

Nurses, including those at the beginning of their careers, such as nurse interns, are often the first to identify potential errors due to their continuous patient contact and active role in care coordination [16]. Nurse interns, by virtue of their transitional status between academia and clinical practice, occupy a unique position in the healthcare system. They are simultaneously learning, adapting, and contributing to patient care in high-stakes environments [17, 18]. However, they are also particularly susceptible to hierarchical pressures, fear of evaluation, and lack of confidence—all of which can discourage them from reporting incidents, especially near misses [19]. Failing to address these barriers risks silencing critical safety voices at the frontline of care.

The willingness of nurse interns to report near-miss events is not merely a behavioral choice; it is shaped by their perceptions of organizational culture, leadership support, psychological safety, and the perceived consequences of reporting [20, 21]. A supportive culture that clearly differentiates between blameworthy acts and human fallibility can significantly enhance interns’ engagement in safety practices. Indeed, when nurse interns perceive that the organization values learning from errors and supports open disclosure, they are more likely to contribute to institutional learning by reporting incidents [22]. Conversely, cultures characterized by punitive responses or lack of feedback can inhibit this behavior and perpetuate a cycle of silence [23].

Studies conducted across various healthcare systems have demonstrated that positive perceptions of Just Culture are associated with increased error reporting, improved teamwork, and reduced moral distress among nurses [24]. Despite the global emphasis on promoting safety culture, little empirical evidence exists from Middle Eastern and North African (MENA) contexts, particularly concerning the experiences and reporting behaviors of nurse interns. In Egypt, where the healthcare system is undergoing gradual reforms toward accreditation and quality improvement, understanding the dynamics of Just Culture and its influence on near-miss reporting among nurse interns is critical. Nurse interns often rotate through high-risk units such as surgery, intensive care, and emergency departments, where near misses are both frequent and underreported [25, 26]. Therefore, addressing their perceptions of Just Culture and evaluating the barriers and facilitators to reporting behavior can provide essential insights for patient safety interventions and organizational policy development.

The present study is guided by the need to bridge this knowledge gap by examining how perceptions of Just Culture influence the willingness of nurse interns to report near-miss events within a university hospital setting in Egypt. It aims to provide evidence that can inform the design of supportive reporting systems, educational curricula, and leadership practices that nurture a transparent and learning-oriented environment. By focusing on nurse interns—an often-overlooked group in safety research—this study contributes to the broader discourse on cultivating a culture of safety and ethical accountability in healthcare institutions. Ultimately, fostering a Just Culture among future nurses may lay the groundwork for long-term improvements in patient safety and care quality across the healthcare continuum.

## Aim of the study

This study aimed to assess the perception of Just Culture among nurse interns and examine its influence on their willingness to report near-miss events within governmental university-affiliated hospitals in Egypt. The study further sought to identify the dimensions of Just Culture that are most strongly associated with reporting behavior and to explore potential demographic or institutional predictors influencing these perceptions.

**Research Questions**

1. What is the level of nurse interns’ perception of Just Culture in Egyptian governmental hospitals?
2. To what extent are nurse interns willing to report near-miss events?
3. Is there a statistically significant relationship between nurse interns’ perception of Just Culture and their willingness to report near-miss events?

## Methodology

### Study design and reporting standards

This study employed a cross-sectional analytical design to examine the relationship between nurse interns’ perceptions of Just Culture and their willingness to report near-miss events. The reporting of this study adheres to the STROBE (Strengthening the Reporting of Observational Studies in Epidemiology) checklist for cross-sectional studies, ensuring transparent description of participants, study size, variables, bias, and statistical methods [27].

### Setting

The study was conducted across five governmental university-affiliated hospitals in Cairo, Egypt: Ain Shams University Hospital (Internal Medicine), Demerdash Surgical Hospital, Pediatric University Hospital, Ahmed Shawky Geriatric Hospital, and the Academic Hospital for Heart Surgery. These hospitals collectively represent a diverse patient population and training environments for nurse interns.

### Participants and sampling

The target population comprised all nurse interns (*N* = 264) enrolled in the structured internship program during the 2024/2025 academic year at the five hospitals. A systematic random sampling technique was applied to each hospital’s intern list. Every *kth* intern was selected (sampling interval k ranged from 2 to 3 depending on hospital size), with proportional allocation across hospitals to reflect the distribution of interns in different service lines. Of the 264 eligible interns approached, 163 consented and completed the study, yielding a response rate of 61.7%. Inclusion criteria were current enrollment in the internship program during the study period. Interns repeating or having previously completed the program were excluded to maintain homogeneity.

### Sample size

Sample size was calculated using G*Power 3.1, assuming a moderate correlation (*r* = 0.30), 95% power, and α = 0.05. The minimum required sample was 138, which was inflated by 15% to account for non-response, giving a target of 163 participants.

### Measures

#### Just Culture Assessment Tool (JCAT)

Perceptions of Just Culture were measured using the Just Culture Assessment Tool (JCAT) developed by Petschonek et al. (2013) [28]. The JCAT consists of 27 items across six domains: balance, trust, openness, quality of reporting process, feedback and communication, and continuous improvement. Responses were rated on a five-point Likert scale (1 = strongly disagree to 5 = strongly agree). The tool was translated into Arabic using forward–backward translation and reviewed by a panel of five bilingual experts. Internal consistency in this study was excellent (Cronbach’s α = 0.91). To further validate the Arabic version, exploratory factor analysis (EFA) was performed, which confirmed the six-factor structure, and confirmatory factor analysis (CFA) in AMOS yielded good model fit indices (χ²/df = 1.89, CFI = 0.95, TLI = 0.94, RMSEA = 0.06, SRMR = 0.05). Factor loadings ranged from 0.62 to 0.84, supporting construct validity.

#### Willingness to report near-miss scale

Willingness to report near misses was assessed using a newly developed scale based on the AHRQ Patient Safety Culture Framework and expert input. The instrument included six dimensions: awareness, skills, attitude, behavior, frequency, and number of near misses reported. Items were scored on a five-point Likert scale, with higher scores reflecting greater willingness. Reliability in this sample was strong (Cronbach’s α = 0.88). EFA supported a four-factor structure (awareness/skills, attitude, behavior, and frequency), which accounted for 67% of variance, and CFA confirmed acceptable model fit (CFI = 0.93, TLI = 0.91, RMSEA = 0.07). The full item set, scoring rubric, and factor loadings are provided in Supplementary File 1.

### Bias and missing data

Potential selection bias was mitigated through proportional allocation across hospitals. Response bias was minimized by ensuring anonymity and voluntary participation. Missing data were minimal (< 2% per item); these were handled using mean imputation within subscales, consistent with prior psychometric practice.

### Statistical analysis

Data were analyzed using SPSS version 26.0 and AMOS version 24.0 (IBM Corp., NY, USA). Descriptive statistics (mean, SD, frequency, percentage) were used to summarize variables. Associations between continuous variables were assessed using Pearson’s correlation. Group comparisons used χ² tests or independent t-tests as appropriate. To identify predictors of willingness to report near misses, multiple linear regression was conducted. Covariates included demographic characteristics (age, gender, marital status), prior training in Just Culture and near-miss reporting, and hospital affiliation (dummy-coded to account for fixed hospital effects). Regression diagnostics confirmed assumptions of linearity, homoscedasticity, normality of residuals, and absence of multicollinearity (all VIF < 2). Statistical significance was set at *p* < 0.05.

### Ethical considerations

The study protocol was reviewed and approved by the Scientific Research Ethics Committee of the Faculty of Nursing at Ain Shams University. Institutional permissions were secured through formal letters issued to hospital administrators. Written informed consent was obtained from all participants prior to data collection. They were assured that participation was voluntary, and that they had the right to withdraw at any time without penalty. Anonymity and confidentiality were strictly maintained, with all responses stored securely and used solely for research purposes. No identifying information was collected. The ethical principles of the Declaration of Helsinki were adhered to throughout the study. Data collectors were faculty research assistants who were not involved in the interns’ supervision or evaluation, ensuring that participation was voluntary and free from any perceived coercion.

## Results

### Demographic characteristics of nurse interns

The study included 163 nurse interns, with a majority aged 23 years or younger (73.0%, mean = 23.3 ± 0.7). Most participants were female (63.8%) and single (82.8%). Over half had received training on Just Culture (54.6%), while fewer (47.2%) had attended training specifically on near-miss reporting. Detailed demographic characteristics are presented in Table 1.

**Table 1:** Demographic characteristics of nurse interns (*n* = 163)

Variable	Category	*n*	%
Age (years)	≤ 23	119	73.0
	> 23	44	27.0
Mean ± SD	23.3 ± 0.7		
Gender	Male	59	36.2
	Female	104	63.8
Marital status	Single	135	82.8
	Married	28	17.2
Training in Just Culture	Yes	89	54.6
	No	74	45.4
Training in Near-Miss	Yes	77	47.2
Reporting	No	86	52.8

### Perception of just culture

Nurse interns’ perceptions of Just Culture were moderate overall (M = 3.6, SD = 0.5). Among the subdomains, continuous improvement (M = 3.9 ± 0.7) and feedback (M = 3.8 ± 0.7) were rated most positively, reflecting the interns’ appreciation of organizational learning and responsiveness. In contrast, balance (M = 3.4 ± 0.7) and the quality of reporting processes (M = 3.5 ± 0.6) received lower ratings, suggesting weaknesses in systemic fairness and reporting infrastructure. These findings are summarized in Table 2.

**Table 2:** Mean scores of just culture and subdomains

Subdomain	Min–Max	Mean ± SD	Median
Feedback	1.7–5.0	3.8 ± 0.7	4.0
Openness	1.0–4.8	3.5 ± 0.7	3.6
Balance	1.6–5.0	3.4 ± 0.7	3.2
Quality of reporting process	1.8–5.0	3.5 ± 0.6	3.6
Continuous improvement	1.2–5.0	3.9 ± 0.7	4.0
Trust	1.8–4.8	3.7 ± 0.6	3.8
**Total Just Culture**	1.9–4.7	**3.6 ± 0.5**	3.6

### Willingness to report near misses

The mean willingness to report near-miss events was also moderate (M = 3.6, SD = 0.5). Awareness (M = 3.9 ± 0.6) and skills (M = 3.6 ± 0.7) were the strongest dimensions, indicating that interns recognized the importance of reporting and felt prepared to do so. However, behavior (M = 3.6 ± 0.5) and attitude (M = 3.5 ± 0.5) scored slightly lower, highlighting a gap between intention and actual reporting practice. Frequency of reporting received the highest score (M = 4.1 ± 0.8), suggesting that when interns did report, they tended to do so consistently. Results are detailed in Table 3.

**Table 3:** Mean scores of willingness to report near misses and subdomains

Subdomain	Min–Max	Mean ± SD	Median
Awareness	2.0–5.0	3.9 ± 0.6	4.0
Skills	1.0–5.0	3.6 ± 0.7	3.7
Behavior	2.3–5.0	3.6 ± 0.5	3.5
Attitude	2.0–4.9	3.5 ± 0.5	3.6
Frequency of reporting	3.0–5.0	4.1 ± 0.8	4.0
**Total Willingness**	2.3–4.8	**3.6 ± 0.5**	3.6

### Correlation between just culture and willingness to report

Pearson’s correlations indicated strong and statistically significant positive relationships between Just Culture and willingness to report. The overall Just Culture score was highly correlated with overall willingness (*r* = 0.68, *p* < 0.001). At the subdomain level, trust (*r* = 0.61) and feedback (*r* = 0.55) were most strongly associated with willingness dimensions, while the quality of reporting process showed no significant relationship. Correlation results are shown in Table 4.

**Table 4:** Correlations between just culture and willingness to report

Variable	Awareness	Skills	Attitude	Behavior	Total Willingness
Feedback	0.49***	0.42***	0.38***	0.46***	0.55***
Openness	0.41***	0.35***	0.39***	0.44***	0.48***
Balance	0.38***	0.33***	0.36***	0.40***	0.43***
Quality	0.15 ns	0.12 ns	0.11 ns	0.10 ns	0.13 ns
Continuous Impr.	0.44***	0.36***	0.37***	0.41***	0.46***
Trust	0.55***	0.52***	0.49***	0.57***	0.61***
**Total JC**	0.62***	0.58***	0.55***	0.63***	0.68*

### Predictors of willingness to report (Overall model)

Multiple linear regression was conducted to identify predictors of willingness to report. The overall model was significant, explaining 55% of the variance (adjusted R² = 0.55, F(7,155) = 28.9, *p* < 0.001). Just Culture score emerged as the strongest predictor (B = 0.70, β = 0.67, *p* < 0.001). Marital status showed a small negative effect (B = − 0.18, β = − 0.13, *p* = 0.018), while affiliation with the pediatric hospital had a modest positive effect (B = 0.05, β = 0.14, *p* = 0.010). Full regression results are presented in Table 5.

**Table 5:** Multiple linear regression predicting willingness to report near misses

Predictor	B	SE	β	95% CI (B)	*p*-value
Constant	1.17	0.23	—	0.71–1.62	< 0.001
Marital status (M)	–0.18	0.08	–0.13	–0.33 – − 0.03	0.018
Pediatric hospital	0.05	0.02	0.14	0.01–0.09	0.010
Just Culture score	0.70	0.06	0.67	0.59–0.82	< 0.001

### Predictors of willingness to report (JCAT subdomain model)

An exploratory regression model including the six JCAT subdomains as simultaneous predictors explained slightly more variance (adjusted R² = 0.57, F(6,156) = 36.8, *p* < 0.001). Trust (B = 0.28, β = 0.33, *p* < 0.001) and feedback (B = 0.22, β = 0.27, *p* = 0.002) were the most influential independent predictors, while balance, openness, continuous improvement, and quality of the reporting process were not statistically significant after adjustment. This highlights the importance of interpersonal trust and organizational responsiveness in shaping interns’ willingness to report near-miss events. Findings are shown in Table 6.

**Table 6:** Regression model with JCAT subdomains as predictors of willingness

Subdomain	B	SE	β	95% CI (B)	*p*-value
Feedback	0.22	0.07	0.27	0.08–0.36	0.002
Openness	0.10	0.08	0.12	–0.06–0.26	0.213
Balance	0.09	0.07	0.11	–0.05–0.23	0.189
Quality	0.04	0.06	0.05	–0.08–0.16	0.512
Continuous Impr.	0.07	0.08	0.09	–0.09–0.23	0.371
Trust	0.28	0.06	0.33	0.16–0.40	< 0.001

## Discussion

The present study examined the influence of Just Culture on nurse interns’ willingness to report near-miss events across five Egyptian university hospitals. The findings revealed moderate mean scores for both Just Culture (M = 3.6, SD = 0.5) and willingness to report near misses (M = 3.6, SD = 0.5). These results underscore the importance of organizational culture in shaping early-career nurses’ reporting behaviors, while also highlighting areas requiring improvement.

### Interpretation of key findings

The study found that trust and feedback were the strongest predictors of willingness to report, consistent with international research demonstrating that organizational responsiveness and fairness are central to encouraging voluntary error disclosure [11, 29, 30]. Importantly, continuous improvement also scored highly (M = 3.9), almost comparable to feedback (M = 3.8). This suggests that interns value not only immediate feedback mechanisms but also perceive broader system-level efforts to learn and improve from reported events. Both dimensions should be emphasized when designing reporting interventions [31–33].

By contrast, balance (M = 3.4) and quality of the reporting process (M = 3.5) were rated lowest, indicating that interns perceive gaps in procedural fairness and system usability. This aligns with earlier evidence suggesting that bureaucratic processes, lack of anonymity, and poor interface design can discourage reporting even in culturally supportive environments [20, 34].

From the perspective of the Theory of Planned Behavior (TPB), interns demonstrated relatively high awareness and skills (attitudes toward reporting were positive), but lower scores for behavior and attitude compared to intention [35]. This gap highlights the classic intention–behavior discrepancy, where positive cognitive and normative orientations do not always translate into consistent action [36]. In TPB terms, while attitudes and subjective norms may be supportive, perceived behavioral control—shaped by usability of reporting systems and clarity of consequences—appears weaker in this cohort. Addressing system barriers and reinforcing psychological safety may help narrow this gap [37].

### Comparison with international benchmarks

The moderate Just Culture mean (3.6) observed here is consistent with scores reported in multi-country studies using the JCAT, which typically range between 3.4 and 3.8 among nursing staff [28, 38]. This indicates that Egyptian nurse interns’ perceptions are broadly comparable to international peers, though still below optimal benchmarks (≥ 4.0) seen in high-reliability organizations [39]. The moderate willingness scores (3.6) are also aligned with findings from studies of early-career nurses in other contexts, underscoring the global challenge of converting intention into actual reporting behavior [40, 41].

### Contextual predictors

Two additional predictors emerged from the regression models. First, pediatric hospital affiliation was positively associated with willingness to report. This may reflect the heightened vigilance and ethical sensitivity required when caring for vulnerable populations, where the stakes of near misses are perceived as higher [25, 31]. However, this effect should be interpreted cautiously, as it may also reflect unmeasured institutional policies or leadership practices unique to the pediatric setting. Second, marital status was negatively associated with willingness, suggesting that married interns may perceive greater risks in reporting or have competing personal and professional pressures. Given the small proportion of married participants (17.2%), this finding should be considered preliminary and warrants further investigation in larger samples.

### Implications for nursing practice

The findings of this study have several important implications for nursing practice, particularly in clinical education, patient safety, and leadership. First, fostering a Just Culture within healthcare settings is critical to enhancing nurse interns’ willingness to report near-miss incidents. Early-career nurses, especially interns, operate in environments where psychological safety and organizational support strongly influence their behavior. Nurse leaders must therefore prioritize the development of a non-punitive culture that values learning from errors and reinforces ethical accountability.

Second, the study highlights the need for structured training programs that go beyond theoretical knowledge and address the emotional, cognitive, and ethical aspects of error reporting. Integrating simulations, reflective debriefings, and peer-led safety discussions into internship programs can help bridge the gap between awareness and reporting behavior.

Third, nurse managers should ensure that reporting systems are transparent, user-friendly, and provide timely feedback. The low perception of the “quality of reporting processes” suggests that system usability is a barrier. Therefore, technological improvements—such as anonymous digital platforms or real-time mobile reporting tools—may enhance reporting frequency and reliability.

Lastly, findings emphasize the importance of leadership visibility and role modeling. Nursing supervisors must actively demonstrate support for error disclosure and provide interns with reassurance that near-miss reporting is a valued contribution to patient care improvement. When junior nurses observe consistent and fair responses to incidents, they are more likely to adopt these behaviors themselves.

### Limitations of the study

Despite its strengths, this study has several limitations that must be acknowledged. First, the cross-sectional design precludes causal inferences. While significant associations were identified between Just Culture and willingness to report near misses, the temporal direction of these relationships cannot be firmly established.

Second, data were collected using self-administered questionnaires, which are subject to social desirability and recall biases. Interns may have overestimated their willingness to report or provided responses they perceived as professionally favorable.

Third, the study was conducted exclusively in university-affiliated governmental hospitals in Egypt. Therefore, the findings may not be generalizable to other clinical settings, such as private hospitals, rural clinics, or different cultural and healthcare systems. Variations in reporting norms, safety culture maturity, and organizational structures across institutions may influence results.

Additionally, the study focused solely on nurse interns. While this group is critical to understanding transitional behaviors, comparisons with more experienced nurses could have added depth to the analysis. Finally, the reliance on perceived willingness to report—as opposed to actual reporting behavior—may limit the ecological validity of the findings.

## Conclusion

This study demonstrates that nurse interns’ willingness to report near-miss events is significantly shaped by their perceptions of Just Culture, with moderate mean scores suggesting that while awareness and intent are present, consistent reporting behaviors remain limited. To strengthen reporting practices, institutions should adopt practical strategies directly linked to key JCAT dimensions: anonymous e-reporting platforms can enhance trust and reduce fear of blame, real-time feedback dashboards can reinforce transparency and demonstrate system learning, leadership walkrounds can promote openness by modeling non-punitive responses, and simulation-based debriefs can normalize error disclosure while building interns’ confidence. By targeting these cultural dimensions—trust, feedback, and openness—organizations can translate positive perceptions into actual reporting behavior, empowering nurse interns to actively contribute to patient safety while laying the foundation for a sustainable culture of fairness and continuous improvement in healthcare settings.

## Supplementary Information

Below is the link to the electronic supplementary material.

Supplementary Material 1

## Data Availability

The datasets used and analyzed during the current study are available from the corresponding author on reasonable request.
