# Supplementary material for: Just culture and its influence on nurse interns’ willingness to report near-miss events: a cross-sectional study in five Egyptian university hospitals
Source: BMC Nurs. 2025 Nov 13;24:1397. doi: 10.1186/s12912-025-03979-w (PMC12616966; doi:10.1186/s12912-025-03979-w)
Supplement: Supplementary file 1 — Supplementary Material 1 [file 12912_2025_3979_MOESM1_ESM.docx]

**Willingness to report near-miss questionnaire**

**Section A:** Current use of Willingness to report near-miss subscale

Please place (√) in front of the suitable response in the task as you practice it.

| **Not at all على الاطلاق** | **Slight extent** **الى حد طفيف** | **Moderate extent**  **الى حد متوسط** | **great extent الى حد كبير** | **Very great extent**  **الى حد كبير جدا** | **Items** | **No** |
| --- | --- | --- | --- | --- | --- | --- |
| 1. **Awareness** | | | | | | |
|  |  |  |  |  | I understand what is meant by the term near miss events. |  |
|  |  |  |  |  | Reporting near miss events improve the quality of my work. |  |
|  |  |  |  |  | I recognize the difference between error and near miss events |  |
|  |  |  |  |  | Application of reporting near miss events is necessary in my work. |  |
| 1. **skills** | | | | | | |
|  |  |  |  |  | I am aware of the process of reporting near miss events. |  |
|  |  |  |  |  | I am able to report near miss events to change clinical practice where I work |  |
|  |  |  |  |  | The event reporting system is easy to use. |  |
| 1. **Behavior** | | | | | | |
|  |  |  |  |  | I believe that reporting near miss events results in the best patient safety practice |  |
|  |  |  |  |  | I am sure that I can implement near miss event report. |  |
|  |  |  |  |  | I am clear about the steps of reporting near miss events. |  |
|  |  |  |  |  | I believe that reporting near miss events takes too much time. |  |
|  |  |  |  |  | I believe that reporting near miss events is difficult. |  |
|  |  |  |  |  | I am confident about my ability to report near miss events in my work. |  |
| 1. **Attitude** | | | | | | |
|  |  |  |  |  | I am willing to report near miss events in my work. |  |
|  |  |  |  |  | reporting near miss events provide structured learning |  |
|  |  |  |  |  | It was required by your supervisor. |  |
|  |  |  |  |  | It was required by your agency |  |
|  |  |  |  |  | reporting near miss events is too simplistic. |  |
|  |  |  |  |  | reporting near miss events is too much paperwork. |  |
|  |  |  |  |  | By reporting near miss events, I'm making the hospital a safer place for the patients. |  |
| 1. **Frequency of Events Reported**   **In your work area/unit, when the following mistakes happen, how often are they reported?** | | | | | | |
| **(1)**  **Never** | **(2)**  **Rarely** | **(3)**  **Sometimes** | **(4)**  **Most of the time** | **(5)**  **Always** | **Items** | **No** |
|  |  |  |  |  | When a mistake is made, but is *caught and corrected before affecting the patient*, how often is this reported? |  |
|  |  |  |  |  | When a mistake is made, but has *no potential to harm the patient*, how often is this reported? |  |
|  |  |  |  |  | When a mistake is made that *could harm the patient*, but does not, how often is this reported? |  |

**SECTION B: Number of Events Reported**

**In the past 6 months, how many event reports have you filled out and submitted?**

| 🞎 a. No event reports | 🞎 d. 6 to 10 event reports |
| --- | --- |
| 🞎 b. 1 to 2 event reports | 🞎 e. 11 to 20 event reports |
| 🞎 c. 3 to 5 event reports | 🞎 f. 21 event reports or more |
